# Outcomes of Patients Treated for Hepatoblastoma with Low Alpha-Fetoprotein and/or Small Cell Undifferentiated Histology: A Report from the Children’s Hepatic Tumors International Collaboration (CHIC)

**DOI:** 10.3390/cancers15020467

**Published:** 2023-01-11

**Authors:** Angela D. Trobaugh-Lotrario, Rudolf Maibach, Daniel C. Aronson, Arun Rangaswami, Beate Häberle, Allison F. O’Neill, Irene Schmid, Marc Ansari, Tomoro Hishiki, Sarangarajan Ranganathan, Rita Alaggio, Ronald R. de Krijger, Yukichi Tanaka, Soo-Jin Cho, Christian Vokuhl, Rebecca Maxwell, Mark Krailo, Eiso Hiyama, Piotr Czauderna, Milton Finegold, James H. Feusner, Marcio H. Malogolowkin, Rebecka L. Meyers, Dolores Lopez-Terrada

**Affiliations:** 1 Department of Pediatric Hematology/Oncology, Providence Sacred Heart Children’s Hospital, Spokane, WA 99204, USA; 2 IBCSG Coordinating Center, 3008 Bern, Switzerland; 3 Department of Pediatric Surgery, University Children’s Hospital Zurich, 8032 Zurich, Switzerland; 4 Division of Pediatric Hematology and Oncology, University of California, San Francisco, CA 94143, USA; 5 Division of Pediatric Surgery, Dr. von Hauner Children’s Hospital, Ludwig-Maximilians-University Munich, 80337 Munich, Germany; 6 Department of Pediatric Oncology, Dana-Farber Cancer Institute, Boston Children’s Hospital and Harvard Medical School, Boston, MA 02215, USA; 7 Department of Pediatric Hematology and Oncology, Dr. von Hauner Children’s Hospital, Ludwig-Maximilians-University Munich, 80337 Munich, Germany; 8 Pediatric Department, Onco-Hematology Unit, University of Geneva Hospitals, 1211 Geneva, Switzerland; 9 Department of Pediatric Surgery, Chiba University Graduate School of Medicine, Chiba 260-8677, Japan; 10 Division of Pathology and Laboratory Medicine, Cincinnati Children’s Hospital Medical Center, Cincinnati, OH 45229, USA; 11 Department of Pathology, Bambino Gesu Children’s Hospital, 00165 Rome, Italy; 12 Department of Pathology, University Medical Center Utrecht, Heidelberglaan 100, 3584 CX Utrecht, The Netherlands; 13 Laboratory for Childhood Cancer Pathology, Princess Maxima Center for Pediatric Oncology, Heidelberglaan 25, 3584 CS Utrecht, The Netherlands; 14 Department of Pathology, Kanagawa Children’s Medical Center, Yokohama 232-0066, Japan; 15 Department of Pathology, University of California, San Francisco, CA 94143, USA; 16 Institute of Pathology, University Hospital Bonn, 53127 Bonn, Germany; 17 Department of Population and Public Health Sciences, University of Southern California, Los Angeles, CA 90032, USA; 18 Department of Pediatric Surgery, Hiroshima University, Hiroshima 734-8551, Japan; 19 Department of Surgery for Children and Adolescents, Medical University of Gdansk, 80-210 Gdansk, Poland; 20 Department of Pathology and Immunology, Baylor College of Medicine Houston, TX 77030, USA; 21 Division of Hematology/Oncology, Children’s Hospital & Research Center Oakland, Oakland, CA 94618, USA; 22 Division of Pediatric Hematology Oncology, University of California-Davis, Davis, CA 95817, USA; 23 Division of Pediatric Surgery, Primary Children’s Hospital, Salt Lake City, UT 84113, USA

**Keywords:** Hepatoblastoma, SCU (small cell undifferentiated), rhabdoid, AFP, *SMARCB1*

## Abstract

**Simple Summary:**

Hepatoblastoma is the most common malignant liver tumor in children. Small cell undifferentiated histology and low alpha-fetoprotein have been previously reported as factors associated with poor prognosis. It is important to exclude rhabdoid tumors (a rare pediatric liver tumor that is difficult to cure) in patients diagnosed with hepatoblastoma with alpha-fetoprotein levels less than 100 ng/mL and in patients with hepatoblastoma with small cell undifferentiated histology since the treatment for these patients is very different. When rhabdoid tumors are correctly diagnosed with testing for loss of *SMARCB1*, neither hepatoblastoma with small cell undifferentiated component nor alpha-fetoprotein less than 100 ng/mL confer poor prognosis.

**Abstract:**

Small cell undifferentiated (SCU) histology and alpha-fetoprotein (AFP) levels below 100 ng/mL have been reported as poor prognostic factors in hepatoblastoma (HB); subsequent studies reported *SMARCB1* mutations in some SCU HBs confirming the diagnosis of rhabdoid tumor. The Children’s Hepatic tumors International Collaboration (CHIC) database was queried for patients with HB who had AFP levels less than 100 ng/mL at diagnosis or were historically diagnosed as SCU HBs. Seventy-three of 1605 patients in the CHIC database were originally identified as SCU HB, HB with SCU component, or HB with low AFP levels. Upon retrospective review, they were re-classified as rhabdoid tumors (*n* = 11), HB with SCU component (*n* = 41), and HB with low AFP (*n* = 14). Seven were excluded for erroneously low AFP levels. Overall survival was 0% for patients with rhabdoid tumors, 76% for patients with HB with SCU component, and 64% for patients with HB with AFP less than 100 ng/mL. Patients with HB with SCU component or low AFP should be assessed for *SMARCB1* mutations and, if confirmed, treated as rhabdoid tumors. When rhabdoid tumors are excluded, the presence of SCU component and low AFP at diagnosis were not associated with poor prognosis in patients diagnosed with HB.

## 1. Introduction

Hepatoblastoma (HB) is associated with elevated levels of alpha-fetoprotein (AFP) at diagnosis and varied histological patterns [1,2,3]. HB with small cell undifferentiated (SCU) histology and AFP less than 100 ng/mL at diagnosis have been previously associated with poor prognosis with survival rates of 24 to 37.5% [4,5,6]. Molecular characterization of SCU HBs revealed that a proportion of HBs with SCU histology are actually primary rhabdoid tumors with characteristic loss-of-function variants or deletions of the *SMARCB1* gene, loss of INI1 [7,8], low AFP levels, and poor outcomes [9]. The availability of immunohistochemical markers to identify loss of INI1 expression in rhabdoid tumors and the 2014 international histologic consensus classification of pediatric liver tumors improved the classification of pediatric liver tumors at diagnosis resulting in more accurate classification and fewer tumors erroneously diagnosed as HB, particularly in patients with AFP less than 100 ng/mL [2]. Recent manuscripts have noted that in patients with HB with SCU histology with retention of INI1 expression, the prognosis does not appear to be unfavorable [10,11]. For patients on AHEP0731 with HB with SCU histology (33 of 35 with retained INI1 expression), the five-year event-free survival (EFS) was 86%, 81%, and 29% for patients with low, intermediate, and high-risk HB (compared with five-year EFS of 87%, 88% and 55% for those with HB without SCU) [10]. 

The Children’s Hepatic tumors International Collaboration (CHIC) was formed by joining pediatric liver tumor specialists from four cooperative groups treating pediatric patients with liver tumors (International Childhood Liver Tumours Strategy Group—SIOPEL, Children’s Oncology Group—COG, Japanese Study Group for Paediatric Liver Tumours—JPLT, German Society for Paediatric Oncology and Haematology—GPOH). Data from 1605 patients treated for hepatoblastoma on trials from these four groups were combined in the CHIC database for analysis of prognostic factors and risk stratification [5,12,13]. For this manuscript, the CHIC database was interrogated to identify patients treated for HB who had an AFP less than 100 ng/mL at diagnosis or who were diagnosed as HB with SCU histology, either as SCU HBs or as HB with small cell component to better understand the prognostic significance of these findings. In addition, patients diagnosed on consensus retrospective review by CHIC pathology reviewers as conclusively having rhabdoid tumors were included in the cohort of patients for comparison.

## 2. Materials and Methods

When enrolled on trial by one of the cooperative groups (SIOPEL, COG, JPLT, GPOH), all 1605 patients in the CHIC database were diagnosed as HB. The CHIC database was queried to identify patients treated for HB with AFP less than 100 ng/mL at diagnosis or histologically classified as SCU HB (based upon initial central review by study group pathologists, by a retrospective CHIC single pathology reviewer, or CHIC consensus review). A central pathology review of tumors from 496 patients was conducted by trial group pathologists. CHIC retrospective consensus review of 599 patients for whom slides were available was performed by 7 international pathologists blinded to the original diagnosis and clinical data. Slides for 16 patients (P9645, *n* = 15; INT0098, *n* = 1) were retrieved for review by one of the CHIC consortia pathologists (DLT) for this report. For patients without Evans staging data in the CHIC database, Evans staging was conducted retrospectively based on a review of clinical data. Given that delayed resection was the primary treatment approach in some of the earlier cooperative group trials, Evans staging inherently favored stage 3 (instead of stages 1 or 2 resected at diagnosis) for patients in those trials. All patients received HB-directed treatment with platinum-based chemotherapy in combination with complete resection when possible. EFS and overall survival (OS) were calculated from the date of enrollment in the trial to the date of the event. Events were defined as relapse, progression, second malignant neoplasm, or death. Patients were removed from analysis if the initial AFP was less than 100 ng/mL and the subsequent level was greater than 10,000 ng/mL. Patients were excluded if they were determined to have erroneously low serum AFP levels presumed to be a result of the “hook” effect. The “hook effect” is due to the measurement of AFP with a one-step simultaneous sandwich immunoassay (as opposed to a two-step immunoassay) which can result in saturation of antibody binding sites by a high AFP concentration with competitive binding to the (measuring) solid phase antibody and subsequent falsely lower reported AFP levels [14,15,16]. This effect can be eliminated with serial dilutions.

## 3. Results

**Figure 1:**
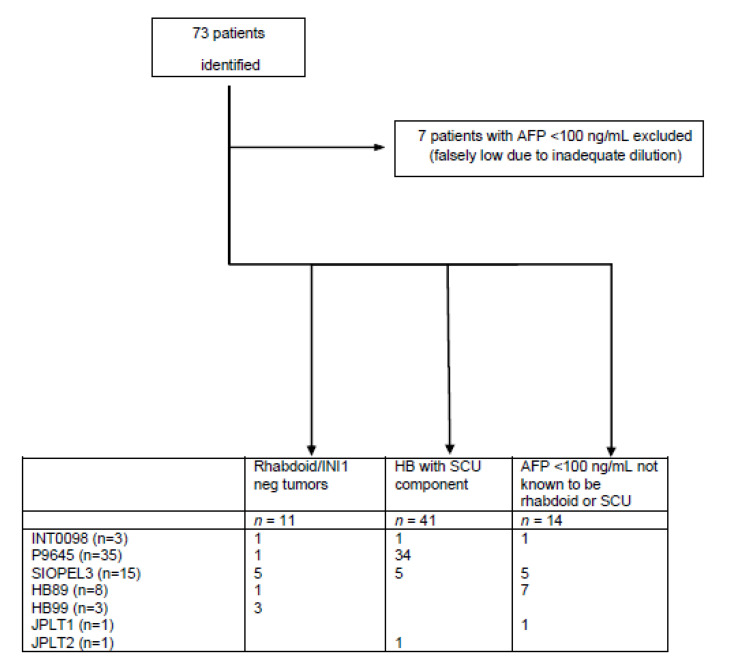
CONSORT statement to identify patients treated for hepatoblastoma who had alpha-fetoprotein levels below 100 ng/mL or who were historically diagnosed as small cell undifferentiated hepatoblastoma. Retrospective CHIC consensus pathology review: 20 patients; Previously known to have loss of *SMARCB1* expression [7]: 1 patient. Histological slides available for review by CHIC pathologist DLT: 16 patients. Trial retrospective review: 4 patients. Trial pathology reports review: 25 patients. AFP, alpha-fetoprotein; HB, hepatoblastoma; SCU, small cell undifferentiated.

From the query of the CHIC database, seventy-three patients were diagnosed as having rhabdoid tumors, HB with AFP less than 100 ng/mL at diagnosis, or HB with SCU component (based upon initial central review by study group pathologists, by a retrospective CHIC single pathology reviewer, or CHIC consensus review). Seven patients were excluded due to erroneously low serum AFP levels presumed to be secondary to the “hook” effect. Sixty-six patients were available for analysis (Figure 1; Table 1 and Table 2) [17,18,19,20,21,22,23]. Tumor slides from 20 patients were available for retrospective consensus review, and 1 patient was previously known to have loss of INI1 expression [7]. Of the 46 patients without CHIC consensus review, histological slides were available for 16 patients enrolled in COG studies P9645 and INT0098 and reviewed by one of the CHIC pathologists [DLT], or trial clinical data (not previously available in the CHIC database) was provided by the cooperative group CHIC contributors [RLM, IS, BH, CV]. Eleven patients were identified as having rhabdoid tumors/loss of *SMARCB1* expression by CHIC consensus review, including 10 with documented loss of *SMARCB1* expression. It is possible in some cases to recognize rhabdoid features and diagnose these tumors without INI1 immunohistochemistry. Other cases are very primitive and undifferentiated with only small cells and require INI1 immunohistochemistry to confirm the diagnosis of rhabdoid tumor. The one patient without documented INI1 loss was able to be recognized as rhabdoid without INI1 immunohistochemistry. Forty-one patients were diagnosed with HB with SCU component upon histological review and did not demonstrate histologic features of rhabdoid tumors. The presence of a predominant SCU component or minor SCU component was assessed by CHIC consensus review; however, it was not possible to accurately quantitate the amount of the SCU component due to the limited tumor sample available for evaluation. SCU was evaluated prospectively on tumor samples from patients enrolled on P9645 alone. It was not reported prospectively in the other trials. Retrospective review for SCU was subsequently conducted by pathologists from SIOPEL and GPOH to assess its prognostic importance. Fourteen patients [including 1 patient with a tumor re-classified as nested epithelial stromal tumor (NEST) upon consensus review] had an AFP less than 100 ng/mL; eight additional patients with AFP less than 100 ng/mL had rhabdoid tumors. AFP at diagnosis was not reported in 38 of the 66 patients analyzed (AFP level was reported in nine patients with rhabdoid tumors, five patients with HB with SCU component, and 14 patients with low AFP). Thirty-seven of the 38 patients without AFP levels at diagnosis were from COG study P9645 (in which AFP at diagnosis was not collected), and one patient was from SIOPEL3. They were included in the analysis given the diagnosis of HB with SCU component.

### 3.1. Rhabdoid Tumors

**Figure 2:**
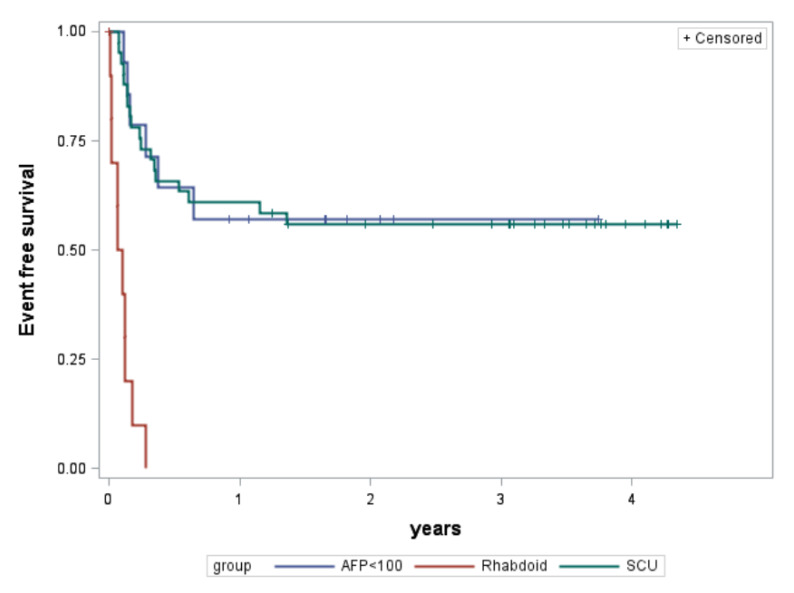
Event-free survival in years by group. AFP, alpha-fetoprotein; HB, hepatoblastoma.

**Figure 3:**
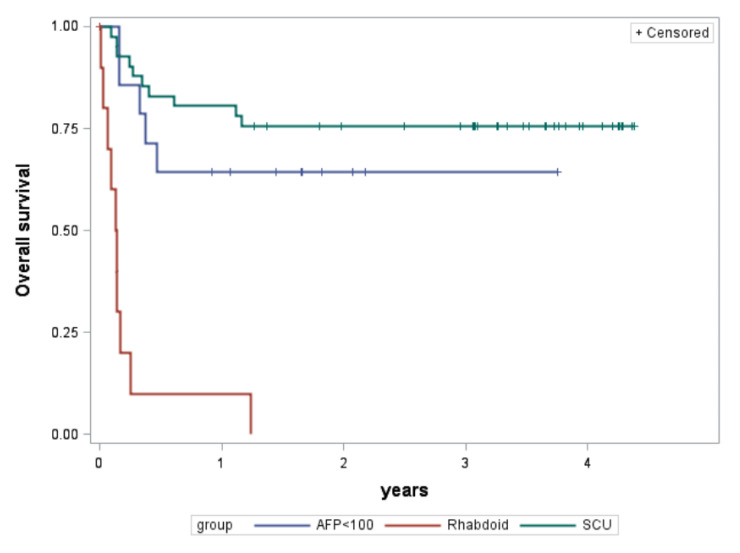
Overall survival in years by group. AFP, alpha-fetoprotein; HB, hepatoblastoma.

Eleven patients were re-classified as having rhabdoid tumors on CHIC retrospective consensus review (including one patient previously confirmed by author MF) [7], with confirmed loss of *SMARCB1* expression by INI1 immunohistochemistry in 10 of the patients (Table 1). Patients with rhabdoid tumors were predominantly male and presented at a younger age at diagnosis. Initial histopathology data reported from the various trials on which these patients were enrolled varied (SCU, *n* = 6; mixed, *n* = 2; not reported, *n* = 3). Two of the three patients without histopathology data in the CHIC database had been later identified by the GPOH authors [IS, BH, CV] on retrospective review to be rhabdoid tumors. These patients presented at advanced stages, and 55% of patients had metastatic disease at diagnosis (Table 1). The AFP at diagnosis was less than 100 ng/mL for all but one patient for which AFP was minimally elevated for age (AFP was not reported in two patients with rhabdoid tumor). Patients were treated with platinum and anthracycline-based chemotherapy regimens (INT0098, *n* = 1; P9645, *n* = 1; SIOPEL3, *n* = 5; GPOH HB89, *n* = 1; GPOH HB99, *n* = 3) and were less likely to have a complete resection of their tumors (Table 2). Three-year EFS and OS in the patients with rhabdoid tumors was 0% (Table 3, Figure 2 and Figure 3).

### 3.2. HBs with SCU Component

Forty-one patients were diagnosed with HB with SCU component and not classified as rhabdoid tumors, and none had documented loss of *SMARCB1* expression by INI1 immunohistochemistry (Table 1). Patients with HB with SCU component were predominantly male and presented at a median age at diagnosis comparable to HB patients overall. HB histologic subtype recorded in the initial trial data varied for these patients (SCU, *n* = 36; embryonal/fetal, *n* = 1; macrotrabecular, *n* = 1; not reported, *n* = 3). Pathology was retrospectively reviewed by CHIC consensus panel (4) or individual CHIC reviewers (16) for 20 of the patients and confirmed to be HB with SCU elements (except one with fetal only), and none of them were classified as rhabdoid tumors. The patient with fetal histology was not excluded given that the histology from trial data was mixed HB with SCU component and limited tumor was available for review to refute the original diagnosis. Patients presented at fairly evenly distributed stages (Table 1). The median AFP at diagnosis was 63,540 ng/mL (range 124 to 380,300) in five patients with data available. AFP data was not reported or available for 36 patients. Patients were treated on platinum and anthracycline-based chemotherapy regimens (INT0098, *n* = 1; P9645, *n* = 34; SIOPEL3, *n* = 5; JPLT2, *n* = 1) with most patients attaining complete resection of their tumors (Table 2). Three-year EFS and OS in the patients with HB with SCU component were 56% and 76%, respectively (Table 3, Figure 2 and Figure 3). Both EFS and OS were significantly better compared to rhabdoid tumors (logrank *p* < 0.0001). Outcomes were comparable to patients without SCU histology when otherwise stratified according to the original CHIC backbone groups (Table 4) [5]. Low numbers preclude *p*-value determination; however, EFS and OS were significantly better than the historical comparison of 37.5% EFS reported by Haas et al. [4].

### 3.3. Low AFP

Fourteen patients had AFP levels less than 100 ng/mL at diagnosis and were not identified as rhabdoid tumors nor HB with SCU component (Table 1). The cohort of patients with low AFP was slightly of male predominance and presented at a median age at diagnosis comparable to all patients with HB (though slightly higher than for patients with HB with SCU component). The median AFP at diagnosis was low at 29 ng/mL (range 0–63). Patients tended to have nonmetastatic tumors that were not resected at diagnosis (Table 1). Pathology slides were available for retrospective consensus review in seven of the 14 patients. Diagnoses on consensus review were epithelial mixed HB (*n* = 4), epithelial pure fetal HB with low mitotic activity (*n* = 2), and NEST (*n* = 1). The initial histologic designation for these patients from trial data varied (epithelial fetal, *n* = 8; epithelial mixed, *n* = 2; poorly differentiated/difficult to classify, *n* = 2; mixed/mesenchymal, *n* = 1; well differentiated, *n* = 1). Three patients with low AFP had PRETreatment EXTent of disease (PRETEXT) I tumors. Patients were treated on platinum and anthracycline-based chemotherapy regimens (INT0098, *n* = 1; SIOPEL3, *n* = 5; JPLT1, *n* = 1; GPOH HB89, *n* = 7) with most patients attaining complete resection of their tumors (Table 2). Three-year EFS and OS in the patients with non-rhabdoid, non-SCU tumors with AFP less than 100 ng/mL were 57% and 64%, respectively (Table 3, Figure 2 and Figure 3). Both EFS and OS were significantly better compared to rhabdoid tumors (logrank *p* < 0.0001). Of the five patients who died, one patient had metastatic disease, one patient had mesenchymal/mixed histology (with no central CHIC consensus review), and three patients had tumors that were poorly differentiated or necrotic following chemotherapy and were difficult to classify (only one of these three had central CHIC consensus review).

## 4. Discussion

Patients with HB with low AFP (below 100 mg/mL) or SCU histology have previously been reported to have poor outcomes. Recent molecular characterization of neoplasms previously designated as SCU HBs has resulted in the reclassification of many of these tumors as rhabdoid tumors given the associated loss of *SMARCB1* expression [7,8] resulting from underlying loss-of-function variants or deletions in the *SMARCB1* gene, sometimes constitutional, characteristic of this group of neoplasms. This has called into question the prognostic relevance of the presence of SCU component and low AFP in patients with HB. 

In the CHIC database, eleven patients with either low AFP or SCU histology were re-classified as rhabdoid tumors. The diagnosis of primary rhabdoid tumors of the liver is currently based on their histopathology in association with their characteristic underlying biology and requires testing for loss of *SMARCB1* expression according to the current classification, which was not available at the time these patients were enrolled in the trial [10]. Patients with rhabdoid tumors of the liver are known to have a poor prognosis with only four of 34 patients surviving in a review of case reports in the literature [9]. Patients from the CHIC database who had rhabdoid tumors treated with HB protocols had a three-year OS of 0%. These patients tended to present with metastatic disease and were unlikely to have primary tumors resected at diagnosis. According to the above-mentioned literature review, the four patients who survived received chemotherapy generally including the agents vincristine, cyclophosphamide, ifosfamide, and doxorubicin, consistent with regimens used for rhabdoid tumors [9,24,25,26,27,28]. 

The definition of SCU component in HB is very subjective and not reproducible (as shown with one of the cases reclassified as fetal). In most cases, the small cell component was interpreted as a mesenchymal or a blastemal component and was focal. Overall survival was 76% for 41 patients with HB with SCU component who were not known to have loss of *SMARCB1* expression (Table 3). Survival was comparable to patients without SCU component when otherwise stratified according to the original CHIC backbone groups (Table 4) [5]. Low numbers of patients in these groups precluded *p*-value determination. The slightly lower outcomes seen in this comparison in patients with HB with SCU component could be due to the histology, but this could also be related to multiple other factors previously identified in the risk stratification analysis of the CHIC database [5]. A multivariate analysis would be necessary to assess the determining factors; however, given the low number of patients, attaining a reliable result is unlikely. 

Patients with AFP less than 100 ng/mL did not have a poor prognosis following the exclusion of tumors reclassified as rhabdoid tumors and those with erroneously low AFP levels due to the “hook” effect. For seven of the 14 patients, no slides were available for central pathology review by CHIC pathologists, and the diagnoses could not be confirmed (including for three of the five patients who died). In addition, some of the trials did not require biopsy at diagnosis, and histologic data was available only following neoadjuvant chemotherapy. It is possible that many patients with AFP less than 100 ng/mL fall into one of two groups: patients with pathology that is actually rhabdoid or other non-HB tumor or patients in subgroups less likely to confer poor outcomes (erroneously low AFP levels due to the “hook” effect or small low-risk PRETEXT I HB tumors). In the present study, three patients had low AFP with PRETEXT I tumors. Of note, for some infants, mildly elevated AFP levels might be appropriate for gestational age [29] and warrant evaluation for rhabdoid, other non-HB tumors, or erroneously low AFP levels due to the “hook” effect.

Some limitations of the current study include the retrospective nature of the data review, incomplete clinical annotation, and limited histological material available for review for some patients (resulting in a small proportion of patients with SCU histology or low AFP at diagnosis for which we could not entirely rule out the diagnosis of rhabdoid tumor). Ongoing and future large, controlled studies including central consensus review, AFP at diagnosis for every patient, and INI1 immunohistochemistry or *SMARCB1* genetic testing for suspected rhabdoid tumor cases would be required to confirm these findings.

## 5. Conclusions

Pediatric patients with liver tumors difficult to classify histologically, with small undifferentiated histology but showing loss of *SMARCB1* expression, should be diagnosed as rhabdoid tumors [30]. These tumors have poor outcomes when treated with HB therapies and should be treated as rhabdoid tumors. Contrary to previous reports, patients with HB with AFP less than 100 ng/mL do not have an inferior prognosis, when rhabdoid and other non-HB tumors are biopsied at diagnosis and ruled out by current histological/immunohistochemical criteria. Given that the presence of SCU component in HB with retention of *SMARCB1* expression does not confer an inferior prognosis, such patients should be treated as HB. It is important to exclude rhabdoid tumors in patients diagnosed with HB with AFP less than 100 ng/mL (or normal for gestational age) and in patients with tumors histologically diagnosed as HB with SCU component, since the treatment for these patients should be very different. Neither the presence of SCU component nor AFP less than 100 ng/mL confer poor prognosis in patients with HB, once rhabdoid tumors are ruled out by immunohistochemical or molecular testing.

## Figures and Tables

**Table 1:** Patient and tumor characteristics by group.

Factor		Rhabdoid (*n* = 11)		SCU (*n* = 41)		Low AFP (*n* = 14)	
Sex	Male Female	9 2	82% 18%	24 17	59% 41%	8 6	57% 43%
Age in years	Median Range	0.71 0.32–1.35		1.45 0.22–4.80		1.80 0.18–10.34	
AFP in ng/mL	Median Range	16 (*n* = 9) 1–756		63,540 (*n* = 5) 124–380,300		29 (*n* = 14) 0–63	
PRETEXT	1 2 3 4 Missing	0 3 7 1	0% 27% 64% 9%	4 16 10 6 5	10% 39% 24% 15% 12%	1 3 8 2	7% 21% 57% 14%
Evans stage	1 2 3 4	0 2 3 6	0% 18% 27% 55%	11 12 5 13	27% 29% 12% 32%	2 2 8 2	14% 14% 57% 14%
Metastatic		6	55%	13	32%	2	14%

SCU, small cell undifferentiated; AFP, alpha-fetoprotein; PRETEXT, PRETreatment EXTent of disease.

**Table 2:** Treatment details by group.

Treatment		Rhabdoid (*n* = 11)	SCU (*n* = 41)	Low AFP (*n* = 14)
# neoadjuvant cycles	Median Range	2 0–6	4 0–12	0 0–7
# adjuvant cycles	Median Range	0 0–5	4 0–12	0 0–6
# patients with cisplatin		9 (82%)	41 (100%)	7 (50%)
# patients with doxorubicin		9 (82%)	4 (10%)	5 (36%)
# patients with carboplatin		7 (64%)	11 (27%)	4 (29%)
# patients with other chemo ^a^		3 (27%)	28 (68%)	1 (7%)
Surgery	Upfront Delayed None/unknown	3 (27%) 1 (9%) 7 (64%)	13 (32%) 10 (24%) 18 (44%)	3 (21%) 6 (43%) 5 (36%)
Result of surgery	Complete Micro residuals Macro residuals Unresectable None/unknown	1 (9%) 2 (18%) 1 (9%) 4 (36%) 3 (27%)	25 (61%) 0 0 3 (7%) 13 (32%)	7 (50%) 1 (7%) 1 (7%) 0 5 (36%)
Metastectomy		1 (9%)	0	0

^a^. One or more of pirarubicin, etoposide, ifosfamide, 5-FU, vincristine, multi-agent chemotherapy. SCU, small cell undifferentiated; AFP, alpha-fetoprotein.

**Table 3:** Event-free survival and overall survival by group.

Outcome		Rhabdoid (*n* = 11)	SCU (*n* = 41)	Low AFP (*n* = 14)
3-year event-free survival	Rate 95% conf.int.	0%	56% 40–70%	57% 28–78%
3-year overall survival	Rate 95% conf.int.	0%	76% 59–86%	64% 34–83%

SCU, small cell undifferentiated; AFP, alpha-fetoprotein.

**Table 4:** Event-free survival comparison with original CHIC dataset, irrespective of histology.

Backbone Group (CHIC Risk Stratification Development [2])	EFS (CHIC)	HB with SCU Component (Current Study), 3-Year EFS
PRETEXT I/II, no mets, AFP > 100	86%	73% (*n* = 15)
PRETEXT III, no mets, AFP > 100	82%	57% (*n* = 7)
PRETEXT IV, no mets, AFP > 100	60%	50% (*n* = 2)
Metastatic disease, AFP > 100	42%	23% (*n* = 13)
		(4 missing due to missing PRETEXT)

CHIC, Children’s Hepatic tumors International Collaboration; EFS, event-free survival; HB, hepatoblastoma; SCU, small cell undifferentiated; PRETEXT, PRETreatment EXTent of disease; AFP, alpha-fetoprotein.

## Data Availability

The data presented in this study are available on request from the corresponding author.

## References

[1] Czauderna P., López-Terrada D., Hiyama E., Häberle B., Malogolowkin M.H., Meyers R.L. (2014). Hepatoblastoma state of the art: Pathology, genetics, risk stratification, and chemotherapy. Curr. Opin. Pediatr..

[2] López-Terrada D., Alaggio R., de Dávila M.T., Czauderna P., Hiyama E., Katzenstein H., Leuschner I., Malogolowkin M., Meyers R., Ranganathan S. (2014). Towards an international pediatric liver tumor consensus classification: Proceedings of the Los Angeles COG liver tumors symposium. Mod. Pathol..

[3] Haas J.E., Muczynski K.A., Krailo M., Ablin A., Land V., Vietti T.J., Hammond G.D. (1989). Histopathology and prognosis in childhood hepatoblastoma and hepatocarcinoma. Cancer.

[4] Haas J.E., Feusner J.H., Finegold M.J. (2001). Small cell undifferentiated histology in hepatoblastoma may be unfavorable. Cancer.

[5] Meyers R.L., Maibach R., Hiyama E., Häberle B., Krailo M., Rangaswami A., Aronson D.C., Malogolowkin M.H., Perilongo G., von Schweinitz D. (2017). Risk-stratified staging in paediatric hepatoblastoma: A unified analysis from the Children’s Hepatic tumors International Collaboration. Lancet Oncol..

[6] De Ioris M., Brugieres L., Zimmermann A., Keeling J., Brock P., Maibach R., Pritchard J., Shafford L., Zsiros J., Czauderna P. (2008). Hepatoblastoma with a low serum alpha-fetoprotein level at diagnosis: The SIOPEL group experience. Eur. J. Cancer.

[7] Trobaugh-Lotrario A.D., Tomlinson G.E., Finegold M.J., Gore L., Feusner J.H. (2009). Small cell undifferentiated variant of hepatoblastoma: Adverse clinical and molecular features similar to rhabdoid tumors. Pediatr. Blood Cancer.

[8] Vokuhl C., Oyen F., Häberle B., von Schweinitz D., Schneppenheim R., Leuschner I. (2016). Small cell undifferentiated (SCUD) hepatoblastomas: All malignant rhabdoid tumors?. Genes Chromosomes Cancer.

[9] Trobaugh-Lotrario A.D., Finegold M.J., Feusner J.H. (2011). Rhabdoid tumors of the liver: Rare, aggressive, and poorly responsive to standard cytotoxic chemotherapy. Pediatr. Blood Cancer.

[10] Trobaugh-Lotrario A., Katzenstein H.M., Ranganathan S., López-Terrada D., Krailo M.D., Piao J., Chung N., Randazzo J., Malogolowkin M., Furman W.L. (2021). Small Cell Undifferentiated Histology Does Not Adversely Affect Outcome in Hepatoblastoma: A Report from the Children’s Oncology Group (COG) AHEP0731 Study Committee. J. Clin. Oncol..

[11] Zhou S., Gomulia B.S., Mascarenhas L., Wang L. (2015). Is INI1-retained small cell undifferentiated histology in hepatoblastoma unfavorable?. Hum. Pathol..

[12] Czauderna P., Häberle B., Hiyama E., Rangaswami A., Krailo M., Maibach R., Rinaldi E., Feng Y., Aronson D., Malogolowkin M. (2016). The Children’s Hepatic tumors International Collaboration (CHIC): Novel global rare tumor database yields new prognostic factors in hepatoblastoma and becomes a research model. Eur. J. Cancer.

[13] Häberle B., Rangaswami A., Krailo M., Czauderna P., Hiyama E., Maibach R., López-Terrada D., Aronson D.C., Alaggio R., Ansari M. (2020). The importance of age as prognostic factor for the outcome of patients with hepatoblastoma: Analysis from the Children’s Hepatic tumors International Collaboration (CHIC) database. Pediatr. Blood Cancer.

[14] Tate J., Ward G. (2004). Interferences in immunoassay. Clin. Biochem. Rev..

[15] Jassam N., Jones C.M., Briscoe T., Horner J.H. (2006). The hook effect: A need for constant vigilance. Ann. Clin. Biochem..

[16] Fernando S.A., Wilson G.S. (1992). Studies of the ‘hook’ effect in the one-step sandwich immunoassay. J. Immunol. Methods.

[17] Ortega J.A., Douglass E.C., Feusner J.H., Reynolds M., Quinn J.J., Finegold M.J., Haas J.E., King D.R., Liu-Mares W., Sensel M.G. (2000). Randomized comparison of cisplatin/vincristine/fluorouracil and cisplatin/continuous infusion doxorubicin for treatment of pediatric hepatoblastoma: A report from the Children’s Cancer Group and the Pediatric Oncology Group. J. Clin. Oncol..

[18] Katzenstein H.M., Chang K.W., Krailo M., Chen Z., Finegold M.J., Rowland J., Reynolds M., Pappo A., London W.B., Malogolowkin M. (2009). Amifostine does not prevent platinum-induced hearing loss associated with the treatment of children with hepatoblastoma: A report of the Intergroup Hepatoblastoma Study P9645 as a part of the Children’s Oncology Group. Cancer.

[19] Zsíros J., Maibach R., Shafford E., Brugieres L., Brock P., Czauderna P., Roebuck D., Childs M., Zimmermann A., Laithier V. (2010). Successful treatment of childhood high-risk hepatoblastoma with dose-intensive multiagent chemotherapy and surgery: Final results of the SIOPEL-3HR study. J. Clin. Oncol..

[20] von Schweinitz D., Byrd D.J., Hecker H., Weinel P., Bode U., Bürger D., Erttmann R., Harms D., Mildenberger H. (1997). Efficiency and toxicity of ifosfamide, cisplatin and doxorubicin in the treatment of childhood hepatoblastoma. Study Committee of the Cooperative Paediatric Liver Tumour Study HB89 of the German Society for Paediatric Oncology and Haematology. Eur. J. Cancer.

[21] Häberle B., Maxwell R., Schweinitz D.V., Schmid I. (2019). High Dose Chemotherapy with Autologous Stem Cell Transplantation in Hepatoblastoma does not Improve Outcome. Results of the GPOH Study HB99. Klin. Padiatr..

[22] Sasaki F., Matsunaga T., Iwafuchi M., Hayashi Y., Ohkawa H., Ohira M., Okamatsu T., Sugito T., Tsuchida Y., Toyosaka A. (2002). Outcome of hepatoblastoma treated with the JPLT-1 (Japanese Study Group for Pediatric Liver Tumor) Protocol-1: A report from the Japanese Study Group for Pediatric Liver Tumor. J. Pediatr. Surg..

[23] Hishiki T., Matsunaga T., Sasaki F., Yano M., Ida K., Horie H., Kondo S., Watanabe K., Oue T., Tajiri T. (2011). Outcome of hepatoblastomas treated using the Japanese Study Group for Pediatric Liver Tumor (JPLT) protocol-2: Report from the JPLT. Pediatr. Surg. Int..

[24] Garcés-Iñigo E.F., Leung R., Sebire N.J., McHugh K. (2009). Extrarenal rhabdoid tumours outside the central nervous system in infancy. Pediatr. Radiol..

[25] Wu X., Dagar V., Algar E., Muscat A., Bandopadhayay P., Ashley D., Wo Chow C. (2008). Rhabdoid tumour: A malignancy of early childhood with variable primary site, histology and clinical behaviour. Pathology.

[26] Ravindra K.V., Cullinane C., Lewis I.J., Squire B.R., Stringer M.D. (2002). Long-term survival after spontaneous rupture of a malignant rhabdoid tumor of the liver. J. Pediatr. Surg..

[27] Scheimberg I., Cullinane C., Kelsey A., Malone M. (1996). Primary hepatic malignant tumor with rhabdoid features. A histological, immunocytochemical, and electron microscopic study of four cases and a review of the literature. Am. J. Surg. Pathol..

[28] Jayaram A., Finegold M.J., Parham D.M., Jasty R. (2007). Successful management of rhabdoid tumor of the liver. J. Pediatr. Hematol. Oncol..

[29] Blohm M.E., Vesterling-Hörner D., Calaminus G., Göbel U. (1998). Alpha 1-fetoprotein (AFP) reference values in infants up to 2 years of age. Pediat.r Hematol. Oncol..

[30] Oda Y., Biegel J.S., Pfister S.M. (2022). Soft Tissue and Bone Tumours, Extrarenal rhabdoid tumour. WHO Classification of Tumours Editorial Board.

